# A New Ticket-Based Authentication Mechanism for Fast Handover in Mesh Network

**DOI:** 10.1371/journal.pone.0155064

**Published:** 2016-05-12

**Authors:** Yan-Ming Lai, Pu-Jen Cheng, Cheng-Chi Lee, Chia-Yi Ku

**Affiliations:** 1 Department of Library and Information Science, Fu Jen Catholic University, New Taipei, Taiwan, 24205, R.O.C.; 2 Department of Computer Science and Information Engineering, National Taiwan University, Taipei, Taiwan, 10617, R.O.C.; 3 Department of Photonics and Communication Engineering, Asia University, Taichung, Taiwan 413, R.O.C.; King Saud University, SAUDI ARABIA

## Abstract

Due to the ever-growing popularity mobile devices of various kinds have received worldwide, the demands on large-scale wireless network infrastructure development and enhancement have been rapidly swelling in recent years. A mobile device holder can get online at a wireless network access point, which covers a limited area. When the client leaves the access point, there will be a temporary disconnection until he/she enters the coverage of another access point. Even when the coverages of two neighboring access points overlap, there is still work to do to make the wireless connection smoothly continue. The action of one wireless network access point passing a client to another access point is referred to as the handover. During handover, for security concerns, the client and the new access point should perform mutual authentication before any Internet access service is practically gained/provided. If the handover protocol is inefficient, in some cases discontinued Internet service will happen. In 2013, Li et al. proposed a fast handover authentication mechanism for wireless mesh network (WMN) based on tickets. Unfortunately, Li et al.’s work came with some weaknesses. For one thing, some sensitive information such as the time and date of expiration is sent in plaintext, which increases security risks. For another, Li et al.’s protocol includes the use of high-quality tamper-proof devices (TPDs), and this unreasonably high equipment requirement limits its applicability. In this paper, we shall propose a new efficient handover authentication mechanism. The new mechanism offers a higher level of security on a more scalable ground with the client’s privacy better preserved. The results of our performance analysis suggest that our new mechanism is superior to some similar mechanisms in terms of authentication delay.

## 1 Introduction

With mobile devices coming to play a bigger and bigger part in our everyday lives, the need for wireless network systems to remain state of the art has become an indispensable urge if the service providers are to stay competitive. The wireless mesh network (WMN) is one of the best-known communication network architectures. It consists of mesh clients and mesh points. Mesh clients can be static hosts (e.g., desktops, servers) or mobile hosts (e.g., smart phones, laptops, and tablets), and they can access the Internet through mesh points. Due to its low cost, large-scale coverage, and high reliability, WMN is widely used nowadays. Several working groups (e.g., IETF) focus their attention on the development of WMN technologies, and corresponding specifications are being standardized (e.g., IEEE 802.12, 802.15 and 802.16).

**Fig 1:**
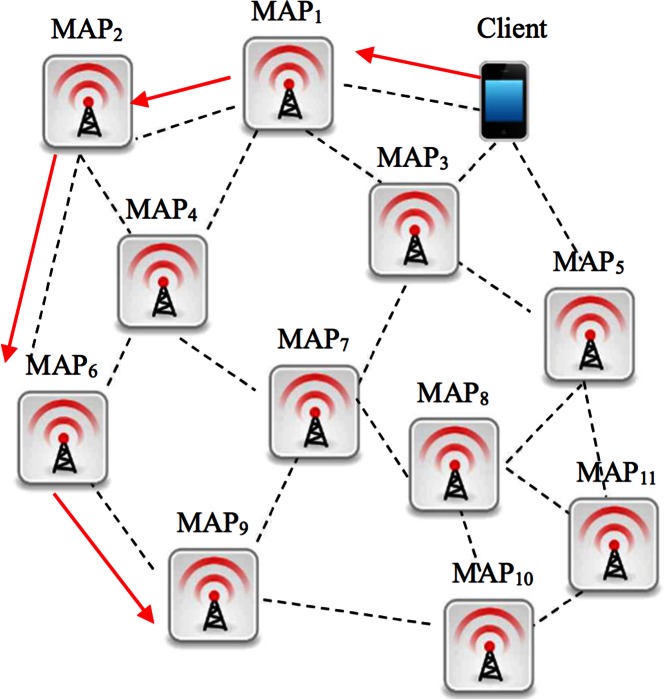
Wireless Mesh Network.

Before accessing the Internet, a client must be authenticated by a mesh access point (MAP). When roaming from a mesh access point to another [1], as illustrated in Fig 1, the client needs to be re-authenticated to receive further Internet services. To keep real-time applications going and thus to offer the best user experience, the overall handover latency should not exceed 50*ms* [2]. However, the current wireless mesh networking standard IEEE 802.16m needs about 1000*ms* to process a full Extensible Authentication Protocol (EAP) for the overlong round trip between the client and the EAP server [3]. To make things worse, this same procedure has to be performed each time when a client moves to a new MAP (e.g. from *MAP*_*1*_ to *MAP*_*2*_) although the current EAP authentication has not yet expired. Obviously, there is plenty of room for improvement.

In order to reduce the latency during client roaming, quite a number of handover authentication protocols have been developed [4–21]. Among them, the earlier works focused on accelerating the full authentication mechanism with the authentication process still having to be repeated every time. Later on, some protocol developers decided that, after the first thorough authentication procedure, neighboring MAPs should pre-recognize the client, and thus the same client can later have a rapid pass by presenting a ticket when entering the realm of a new MAP. These ticket-based protocols mainly fall into three categories, which are handover single authentication, group key authentication, and broadcast authentication. Details of different types of ticket-based protocols will be elaborated in Section 2.

Recently, Li et al. proposed a fast handover authentication mechanism based on tickets for mesh network [22]. In spite of the efficiency and convenience it brings, Li et al.’s mechanism still has some weaknesses. In this paper, we shall present an efficient and secure authentication mechanism we have developed to solve the problems that trouble Li et al.’s mechanism. The main contributions of this paper are as follows.

1. Distinct from the existing handover authentication schemes, the proposed new scheme saves the trouble of digital signature computations on the client side, which significantly reduces the computation cost and handover latency, making the scheme especially suitable for applications where the clients have mobile devices with limited computation power.
2. Unlike most existing handover authentication schemes, the proposed new scheme is applicable when the MAPs are not equipped with high-quality TPDs. Besides, our new scheme can keep another MAP from using the broadcast ticket to pretend to be the current MAP, and therefore there is absolutely no room for the domino effect to happen. In other words, our new scheme is not only more scalable but more secure.
3. In the proposed new scheme, privacy is well preserved, and the authentication imposes negligible overhead.

In this paper, we review Li et al.’s scheme and propose an improvement to refine the approach and make it become applicable. The rest of this paper is organized as follows. Section 2 reviews various ticket-based handover protocols and briefly introduces the basic underlying concepts. Section 3 gives some preliminaries that are to be used throughout this paper. Then, in Section 4, we review and analyze the Li et al.’s scheme. After that, we present an improved scheme in Section 5, followed by a security analysis and a performance analysis of the proposed scheme in Section 6 and Section 7, respectively. Finally, the conclusion will be in Section 8.

## 2 Related Work

Instead of accelerating the full authentication mechanism while repeating the authentication procedure every time, some protocols based on the Kerberos-style ticket [15] have been proposed. The main idea behind these methods is to reduce the authentication latency during the handover process by using a symmetric encrypted ticket. Only certified MAPs own the legal symmetric key and can generate a legal ticket for a verified client. For this reason, any client can submit a legal ticket to prove he/she has passed the authentication procedure with another certified MAP. This way, a legal MAP can simply handover authenticate a verified client. That means a client can wander all over the place receiving non-stop Internet service as long as the ticket has not expired yet. Based on the different mutual authentication mechanisms between client and MAP, we can mainly divide ticket-based authentication schemes into three types: single authentication, group key authentication, and broadcast authentication.

### 2.1 Single Authentication

In ticket-based handover by single authentication [13, 18, 19], it is assumed that each MAP has the pre-stored symmetric key shared among neighboring MAPs. Before a client steps off the coverage of some specific MAP (e.g. *MAP*_*1*_), the client submits a handover single to *MAP*_*1*_ to inform it as to which MAP (e.g. *MAP*_*2*_) he/she will move to. Upon receiving the signal, *MAP*_*1*_ uses *Key*_*MAP1-MAP2*_ to generate a ticket and send this ticket to *MAP*_*2*_. When the client arrives at *MAP*_*2*_, *MAP*_*2*_ can verify the client simply by using the ticket generated by *MAP*_*1*_. However, it will get very confusing when the MAPs within the network are situated in a complicated way [23], for in that case it might not be very clear which MAP is the so-called *MAP*_*2*_ that the client is moving on to.

### 2.2 Group Key Authentication

In ticket-based handover by group key authentication [6–10, 15, 18, 21], the authentication, authorization, and accounting (AAA) server sets up a multi-MAP group, and pre-distributes a private Multi-MAP Group Key (MGK) to each MAP in the group. Thus, a client does not need to inform *MAP*_*1*_ which MAP he/she will move to. *MAP*_*1*_ can use the general MGK to encrypt a ticket and then send it to the client. When the client arrives at *MAP*_*2*_, he/she can readily submit the ticket to *MAP*_*2*_ and get the service. This design based on a single group key, however, is neither secure nor scalable in large-scale mesh networks. To ensure the security of the single group key, each MAP in the group should be a high-quality TPD [24], assuming that they are secure against any compromise attempt in any circumstances. Unfortunately, this is too high a ground to reach [14]. In addition, ticket-based handover by group key authentication will not be an option when any MAP in the group might be malicious.

### 2.3 Broadcast Authentication

In ticket-based handover by broadcast authentication [4, 6, 14, 16, 17, 19], the AAA server maintains every MAP and its neighbors’ locations. When a client is about to leave a MAP, the AAA server will send tickets to all the neighboring MAPs over a secure channel. However, this means there will be a very long latency time because the AAA server is normally many hops away from the client [3, 19]. To solve this problem, Li et al. proposed a fast handover authentication mechanism based on ticket broadcast for mesh network [22]. In their scheme, the task of pre-sending tickets is forwarded from the AAA server to the current MAP (e.g. *MAP*_*1*_). In other words, when the client is about to leave, *MAP*_*1*_ can use a pre-established symmetric key to generate tickets and send corresponding tickets to its neighbors (e.g. *MAP*_*2*_, *MAP*_*3*_, and *MAP*_*4*_). This way, the ticket pre-distribution is completed right between the current MAP and its neighbors, only one hop across. However, in Li et al.’s scheme there are some leaks such as sending expiration time and date in plaintext and pre-sending the same ticket to all the neighboring MAPs. Later in Section 4 we shall give a thorough review of Li et al.’s scheme and detail the leaks we have found.

## 3 Preliminaries

In this section, we present some models and tools commonly used in this field. They include the trust model, different types of tickets, and elliptic curve cryptography (ECC).

### 3.1 Trust Model

**Fig 2:**
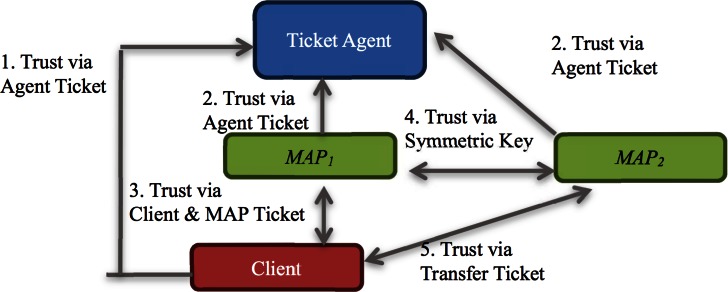
Trust model.

The trust model is illustrated in Fig 2. A ticket agent (TA) is a trusted third party who generates and manages various types of tickets in a mesh network. The following are the elements shown in Fig 2:

- TA—mesh access points (MAPs): The mutual trust is based on public key cryptography and is built when a MAP requests a MAP ticket from TA. In response, TA embeds a digital signature in the MAP ticket to make MAPs believe the ticket was created by TA.
- TA—client: The mutual trust is based on public key cryptography and is built when a client requests a client ticket from TA. In response, TA embeds a digital signature in the client ticket to make the client believe the ticket was created by TA.
- MAP—client: The mutual trust relationship between a client and its home MAP is built through their respective client ticket and MAP ticket, which will be elaborated later.
- MAP_1_—MAP_2_: Any two neighboring MAPs build up mutual trust via symmetric key certificates. This trust allows a client to roam among different MAPs in a mesh network.

Please note that technically both the MAP ticket and the client ticket can be obtained before a client even joins a mesh network, so this portion of time consumption in public key operations should not count as part of the authentication process, which adds to the efficiency of the authentication protocol.

### 3.2 Different Types of Tickets

Three types of tickets are used in this paper: client ticket, MAP ticket, and transfer ticket. They are needed for mutual authentication between a client and a MAP when the client logs into the network or roams from a MAP to another. Before looking any further into the details of the three types of tickets, let’s check out the notations listed in Table 1 below.

**Table 1:** Notation table.

Notation	Description
*C*	Client
*M*	Mesh access point (MAP)
*A*	Ticket agent
*P*_*x*_	Public key assigned to entity *x*
*N*_*x*_	A nonce generated by entity *x*
*Sig*_*x*_	Digital signature of entity *x*
*E*_*Px*_(*m*)	Encryption of the message m by entity *x*’s public key
*D*_*Px*_(*m*)	Decryption of the message m by entity *x*’s public key
*E*_*K*_(*m*)	Encryption of the message m by a shared key *K*
*D*_*K*_(*m*)	Decryption of the message m by a shared key *K*
*K*_*MAC*_	The key used to produce a message authentication code
*V*_*KMAC*_(*m*)	Message authentication code (MAC) of message m in combination with a secret shared key *K*_*MAC*_ [25]
*f* (*m*)	pseudo-random number generation function applied to message *m*
*H*(*m*)	Collision-free one-way hash function applied to message *m* [26]
||	A concatenation operation

#### 3.2.1 Client tickets

The client ticket (*T*_*C*_) enables a client to gain a MAP’s trust. The MAP (e.g. *MAP*_*1*_) can verify the client by checking the client ticket to see if the ticket was really issued by TA.

A client ticket T_C_ typically includes the following elements:

$$T_{C}=\;\left\{I_{C},I_{A},\tau_{exp\_C},P_{C},{Sig}_{A}\right\}$$

- *I*_*C*_: ID of the client who keeps this ticket.
- *I*_*A*_: ID of TA who generated this ticket.
- *τ*_*exp_C*_: expiry time of *T*_*C*_. When this ticket expires, the client needs to re-request a new ticket from TA.

#### 3.2.2 MAP tickets

The MAP ticket (*T*_*M*_) enables a MAP to gain a client’s trust. The client (e.g. *C*) can verify the MAP (e.g. *MAP*_*1*_) by checking the MAP ticket to see if the ticket was truly issued by TA.

A typical MAP ticket *T*_*M*_ includes the following elements:

$$T_{M}=\;\left\{I_{M},I_{A},\tau_{exp\_M},P_{M},{Sig}_{A}\right\}$$

- *I*_*M*_: ID of the MAP who keeps this ticket.
- *I*_*A*_: ID of TA who generated this ticket.
- *τ*_*exp_M*_: expiry time of *T*_*M*_. When this ticket expires, the MAP needs to re-request a new ticket from TA.

#### 3.2.3 Transfer tickets

When a client *C* starts to access the mesh network, he/she needs to exchange his/her client ticket for the nearest MAP’s (e.g. MAP *M*_*1*_) MAP ticket to perform mutual login authentication. If the authentication phase is a success, *M*_*1*_ issues a transfer ticket to *C* and becomes the home MAP of *C*. The transfer ticket helps to construct trust relationship between a foreign MAP (e.g. MAP *M*_*2*_) and *C*. When *C* roams to *M*_*2*_, he/she submits the transfer ticket to *M*_*2*_ for handover authentication. With the transfer ticket, the client *C* can prove to *M*_*2*_ that he/she has been authenticated by *M*_*1*_. Thus, *M*_*2*_ can run a simple authentication process to verify *C*.

The elements of a typical transfer ticket *θ*_*C*_ include:

$$\theta_{C}=\;\{I_{C},I_{M},I_{A},\tau_{exp\_\theta },V_{KMAC}(I_{C},I_{M},I_{A},\tau_{exp\_\theta })\;\}$$

- *I*_*C*_: ID of the client who obtained this ticket.
- *I*_*M*_: ID of the home MAP who generated this ticket.
- *I*_*A*_: ID of TA who issued *I*_*C*_’s client ticket.
- *τ*_*exp_θ*_: expiry time of *θ*_*C*_ which is determined by its issuer’s policy. When this ticket expires, the client needs to re-select a MAP to be the home MAP and re-login to obtain a new transfer ticket. Before that, the client can roam past MAP after MAP with ease.

### 3.3 Elliptic Curve Cryptography (ECC)

Elliptic curve cryptography (ECC) was created by Neal Koblitz and Victor Miller [27] in 1985, and it has been widely used nowadays [20, 28, 29]. Suppose *G* is a group of *p* members, where *p* is a large prime number. Let {*a*, *b*}**∈** Z^*^_n_ be such that 4*a*^3^ +27*b*^2^ ≠ 0 (mod *p*) in *G*. The set *E*(*G*) consists of all points (*x*, *y*)**∈** *G* that satisfy the equation *y*^2^ = *x*^3^ +*ax* +*b*, together with a special point *O*, namely the point at infinity. Then, let’s select a *q*-order subgroup *G*_*q*_ of the additive group of points over *E*(*G*) and choose an arbitrary generator *P* of *G*_*q*_. According to the Elliptic curve discrete logarithm problem (ECDLP), given *mP* **∈** *E*(*G*) and *P* **∈** *G*_*q*_ for an unknown *m* **∈** Z^*^_n_, it is intractable to find *m*. Finally, we preload each client and MAP with the public system parameters {*p*, *q*, *E*(*G*), Z^*^_n_, *P*}.

## 4 Li et al.’s Authentication Protocols

**Fig 3:**
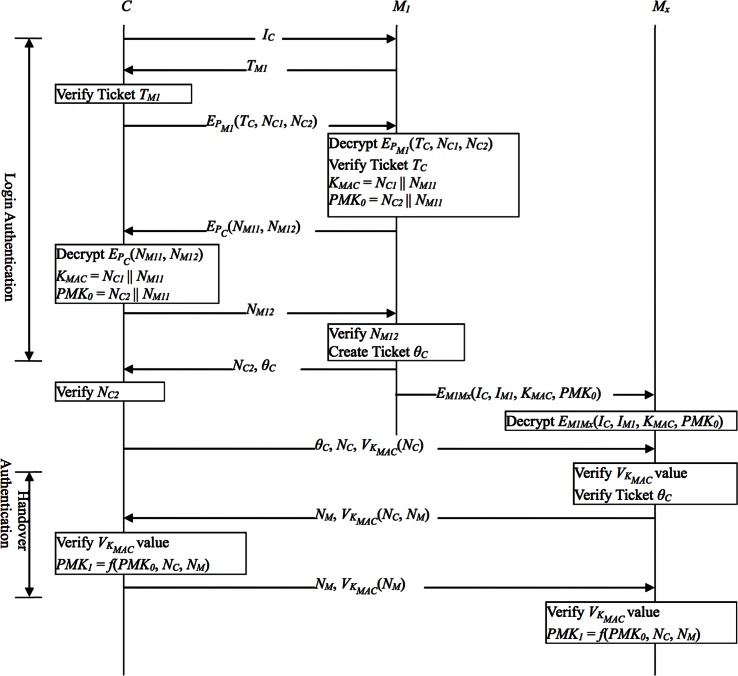
Li et al.’s authentication protocols.

In this section, we shall review Li et al.’s authentication protocols and present our analysis of their protocols. In Li et al.’s scheme [22], there are two distinct authentication protocols: initial login authentication protocol (LAP) and handover authentication protocol (HAP), as shown in Fig 3. These authentication protocols follow a key hierarchical structure similar to that in IEEE 802.11i. A pairwise master key (PMK) is created during the authentication process, and then a pairwise transient key (PTK) and a group transient key (GTK) are derived from the PMK. Because a typical mobile device has limited computation power, the number of message exchanges and that of public key operations should be kept down.

### 4.1 The Login Authentication Protocol (LAP)

Assume that the client and the MAP have respectively obtained a client ticket and a MAP ticket from TA. Now the pair of client and MAP submit tickets to each other for mutual authentication. The steps are as follows:

1. When a client *C* starts to access the mesh network, he/she broadcasts a request message containing his/her ID number to the neighboring MAPs.
2. Assuming MAP *M*_*1*_ receives the request message, *M*_*1*_ replies with a message containing its MAP ticket *T*_*M1*_ to inform *C* of its presence. After receiving *T*_*M1*_, *C* verifies *Sig*_*A*_. If the verification fails, *C* ignores this ticket. Otherwise, *C* checks the *τ*_*exp_M*_ of *T*_*M1*_ and determines whether or not the *τ*_*exp_M*_ has expired.
3. If the above verifications are successful, *C* extracts *M*_*1*_’s public key *P*_*M1*_ from *T*_*M1*_. Then *C* encrypts ticket *T*_*C*_ along with two nonces *N*_*C1*_ and *N*_*C2*_ by using *P*_*M1*_, and sends the encrypted message to *M*_*1*_. Upon receiving the encrypted message, *M*_*1*_ uses its own private key to decrypt the message and then verifies *Sig*_*A*_ in *T*_*C*_. If the verification fails, *M*_*1*_ ignores this ticket. After that, *M*_*1*_ checks the *τ*_*exp_C*_ of *T*_*C*_ and determines whether or not it has expired.
4. If the above verifications are successful, *M*_*1*_ extracts *C*’s public key *P*_*C*_ from *T*_*C*_. Then uses *P*_*C*_ to encrypt two nonces *N*_*M11*_ and *N*_*M12*_. After that, *M*_*1*_ sends the encrypted message to *C* and calculates their shared MAC key *K*_*MAC*_ = *N*_*C1*_||*N*_*M11*_ and pairwise master key *PMK*_*0*_ = *N*_*C2*_||*N*_*M11*_. Upon receiving the message, *C* decrypts it by using his/her private key to obtain *N*_*M11*_ and *N*_*M12*_. Then, *C* calculates their shared MAC key *K*_*MAC*_ = *N*_*C1*_||*N*_*M11*_ and pairwise master key *PMK*_*0*_ = *N*_*C2*_||*N*_*M11*_. Due to the public-key cryptography, the security of nonces *N*_*C1*_, *N*_*C2*_, *N*_*M11*_, and *PMK*_*0*_ is ensured.
5. After that, *C* sends *N*_*M12*_ to *M*_*1*_. Upon receiving this message, *M*_*1*_ verifies *N*_*M12*_ by checking if it matches the value provided by *M*_*1*_ itself earlier. If *N*_*M12*_ does not check out, *M*_*1*_ ignores this message.
6. To complete the login authentication protocol, *M*_*1*_ generates a transfer ticket *θ*_*C*_ and sends *N*_*C2*_ and *θ*_*C*_ to *C*. Upon receiving this message, *C* verifies *N*_*C2*_ by checking if it matches the value provided by *C* himself/herself earlier. If the *N*_*C2*_ value does not check out, *C* ignores this message.

This concludes the login authentication protocol. The pairwise master key *PMK*_*0*_ can then be used to encrypt messages between the two parties. In the meantime, *C* can use this transfer ticket *θ*_*C*_ to legally roam from *MAP*_*1*_ to another MAP in the network.

### 4.2 The Handover Authentication Protocol (HAP)

To support fast handover for clients roaming from *M*_*1*_ to another MAP, *M*_*1*_ should pre-distribute the keys shared with *C* to all the neighboring MAPs by broadcast. Throughout the network, it is assumed that each MAP has established symmetric shared keys with all its neighboring MAPs. After successfully authenticating *C*, *M*_*1*_ encrypts *I*_*C*_, *I*_*M1*_, key *K*_*MAC*_, and the pairwise master key *PMK*_*0*_ shared with *C* by using the key *M*_*1*_ shares with its neighbor *M*_*x*_, and then *M*_*1*_ sends the encrypted message to *M*_*x*_. Upon receiving the message, *M*_*x*_ decrypts it and extracts *K*_*MAC*_ and *PMK*_*0*_ to prepare for future authentication with *C*. The above computations are performed by MAPs, so there is no extra burden laid on the client’s side. When *C* leaves *M*_*1*_ and visits *M*_*x*_, he/she executes the following handover authentication protocol:

1. Client *C* submits his/her transfer ticket *θ*_*C*_, a new number *N*_*C*,_ and Message authentication code *V*_*KMAC*_(*N*_*C*_) to the foreign MAP *M*_*x*_. Upon receiving this message, *M*_*x*_ verifies the correctness of *V*_*KMAC*_(*N*_*C*_) by using *K*_*MAC*_ received from the home MAP *M*_*1*_. If the verification turns out positive, *M*_*x*_ checks *τ*_*exp_θ*_ and the MAC value in *θ*_*C*_ to verify *θ*_*C*_’s validity. Of all clients, only *C* has the knowledge of *K*_*MAC*_ and can generate a valid pair (*N*_*C*_, *V*_*KMAC*_(*N*_*C*_)). This enables the protocol to resist forgery attacks.
2. If the above verifications are successful, *M*_*x*_ sends a nonce *N*_*M*_ and a message authentication code *V*_*KMAC*_(*N*_*C*_, *N*_*M*_) to *C*. After checking out the received message, *C* produces a new pairwise master key *PMK*_*1*_ = *f*(*PMK*_*0*_, *N*_*C*_, *N*_*M*_) for *M*_*x*_. Then *C* sends *N*_*M*_ and *V*_*KMAC*_(*N*_*M*_) to *M*_*x*_ to inform *M*_*x*_ he/she has successfully constructed *PMK*_*1*_.
3. Upon receiving *N*_*M*_ and *V*_*KMAC*_(*N*_*M*_), *M*_*x*_ verifies *V*_*KMAC*_(*N*_*M*_). Since *C* is the only client that has *K*_*MAC*_, a correct *V*_*KMAC*_(*N*_*M*_) proves the identity of *C*. If the verification is successful, *M*_*x*_ also computes *PMK*_*1*_. This concludes the handover authentication process.

### 4.3 Analysis of Li et al.’s authentication protocols

There are, unfortunately, some security flaws in Li et al.’s authentication protocols. For one, the expiration time and date of the transfer ticket *θ*_*C*_ are stored in plaintext. *C* can forge it and re-generate a matched MAC value to illegally extend validity. Secondly, the plaintext *θ*_*C*_ makes it possible for an adversary to track down a specific client, for *θ*_*C*_ involves *I*_*C*_, and *I*_*C*_ is rarely changed. Thirdly, Li et al.’s protocols require that all MAPs should be equipped with high-quality TPDs so that the system can withstand physical attacks. In Li et al.’s design, MAP *M*_*1*_ pre-sends the same ticket to all its neighboring MAPs even though the client may probably move to only one of them (i.e. MAP *M*_*2*_) but not the rest (i.e. MAP *M*_*3*_ and MAP *M*_*4*_). The latter MAPs, however, can still decrypt the ticket and obtain the same secret information (e.g., PMK). This increases security risks. Moreover, the domino effect [6] can also be a problem. The current pairwise master key (PMK) is generated by using the previous PMK along with some public information. Once some MAP *M*_*n*_ has obtained an old PMK, it can track down the current PMK at any time and even disguise as the client to communicate with other MAPs. In other words, once a MAP is compromised, all the MAPs directly or indirectly connected to it will also be affected, and so the whole security system will collapse.

## 5 The Proposed Authentication Protocols

**Fig 4:**
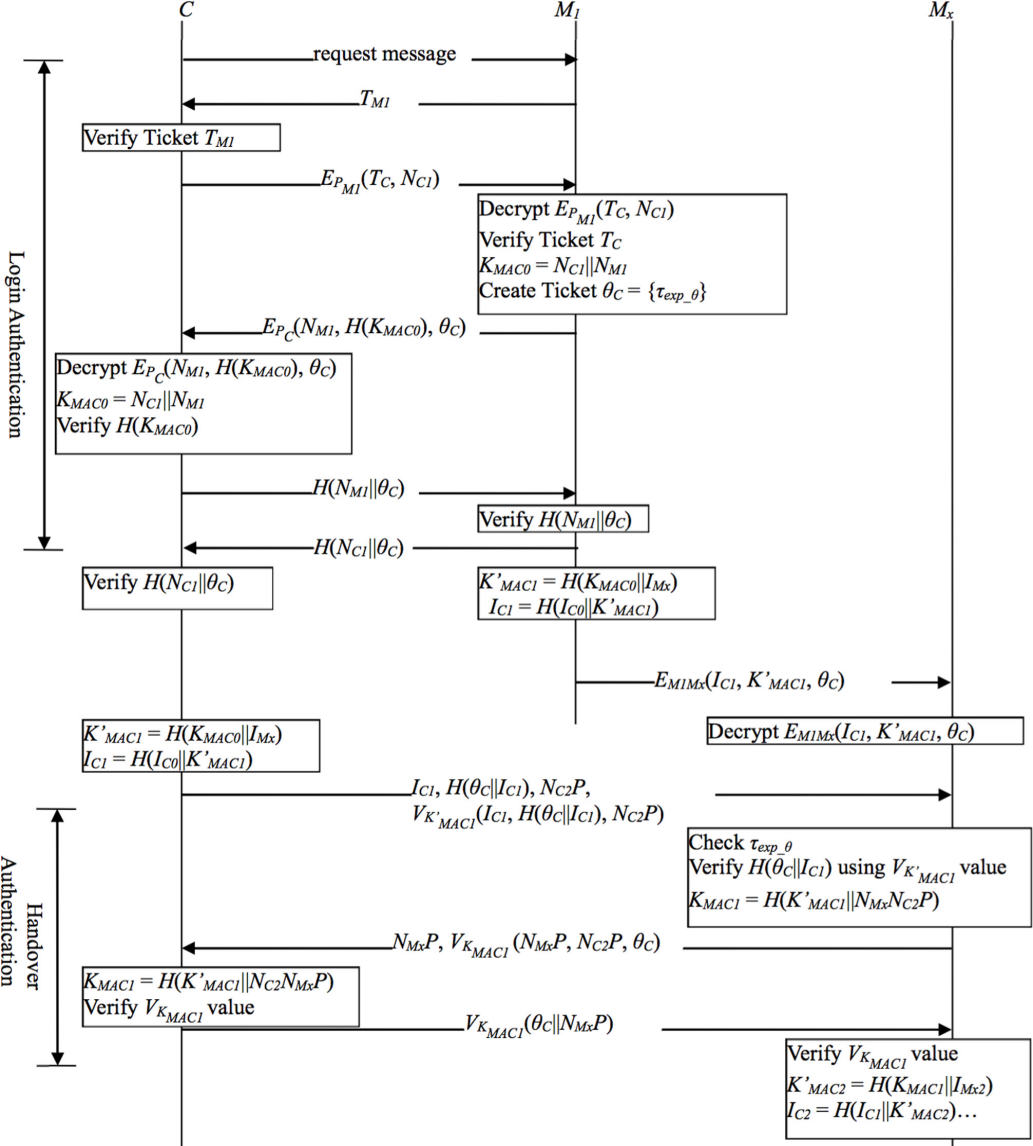
The proposed authentication protocols.

To solve above problems, we shall present an efficient and secure authentication protocols as shown in Fig 4. Furthermore, the proposed protocols preserve clients’ privacy well and only imposes negligible overhead. In the design of proposed protocols, TA is in charge of not only ticket issuing but also ECC public parameter management (e.g., {*p*, *q*, *E*(*G*), Z^*^_n_, *P*} as in Section 3.3).

### 5.1 The Login Authentication Protocol (LAP)

As is stated in the preliminaries section, our login authentication protocol runs upon the assumption that the client and the MAP have respectively obtained a client ticket and a MAP ticket from TA. Now the client-MAP pair submit tickets to each other to do mutual authentication as follows.

1. When a client *C* starts to access the mesh network, he/she broadcasts a request message to the nearby MAPs.
2. Assuming that MAP *M*_*1*_ replies with a message containing its MAP ticket *T*_*M1*_ to inform *C* of its presence. Upon receiving *T*_*M1*_, *C* verifies *Sig*_*A*_ of *T*_*M1*_. If *Sig*_*A*_ fails the verification, *C* ignores this *T*_*M1*_; otherwise, *C* checks *τ*_*exp_M*_ of *T*_*M1*_.
3. If the above verifications are successful, *C* extracts *M*_*1*_’s public key *P*_*M1*_ from *T*_*M1*_. Then *C* encrypts his/her ticket *T*_*C*_ and a nonce *N*_*C1*_ using *P*_*M1*_, and sends the encrypted message to *M*_*1*_. Upon receiving the message, *M*_*1*_ decrypts it and verifies *Sig*_*A*_ in *T*_*C*_. If the verification fails, *M*_*1*_ ignores *T*_*C*_. Otherwise, *M*_*1*_ checks *τ*_*exp_C*_ of *T*_*C*_.
4. If the above verifications are successful, *M*_*1*_ calculates the shared MAC key *K*_*MAC0*_ = *N*_*C1*_||*N*_*M1*_, creates a transfer ticket *θ*_*C*_ = *τ*_*exp_θ*_, and extracts *C*’s public key *P*_*C*_ from *T*_*C*_, where *N*_*M1*_ is a nonce chosen by *M*_*1*_. Then *M*_*1*_ encrypts *N*_*M1*_, *H*(*K*_*MAC0*_), and *θ*_*C*_ using *P*_*C*_, and sends the encrypted message to *C*. Upon receiving the message, *C* decrypts it to obtain *N*_*M1*_, calculates the shared MAC key *K*_*MAC0*_ = *N*_*C1*_||*N*_*M1*_, and verifies *H*(*K*_*MAC0*_). Note that we do not produce any extra pairwise master key PMK, so the bandwidth and key generation cost can both be reduced. In the proposed protocol, we set the MAC key or its derivatives as the session key between *C* and *M*_*1*_. Thus, in the following steps, we only focus on the passing and using of the MAC key.
5. Then *C* sends *H*(*N*_*M1*_||*θ*_*C*_) to *M*_*1*_ to prove that the decryption is successful. Upon receiving this message, *M*_*1*_ checks to see if *H*(*N*_*M1*_||*θ*_*C*_) matches the value owned by itself. If the received *H*(*N*_*M1*_||*θ*_*C*_) does not check out, *M*_*1*_ ignores this message.
6. To complete LAP, *M*_*1*_ returns *H*(*N*_*C1*_||*θ*_*C*_) to *C*. Upon receiving this message, *C* checks to see whether the received *H*(*N*_*C1*_||*θ*_*C*_) matches the value owned by himself/herself. If the value does not match, *C* ignores this message.

This concludes LAP. The value *K*_*MAC0*_ will then be used to encrypt messages between *C* and *M*_*1*_. On the other hand, C can use *K*_*MAC0*_ along with the transfer ticket *θ*_*C*_ to prove his/her legality and roam from *M*_*1*_ to another MAP within the mash network.

### 5.2 The Handover Authentication Protocol (HAP)

To support fast handover for clients roaming from *M*_*1*_ to another MAP, *M*_*1*_ should pre-share some information to all its neighboring MAPs. Instead of broadcasting, we decide to take another route and construct a corresponding temporary MAC key *K’*_*MAC1*_ = *H*(*K*_*MAC0*_||*I*_*Mx*_) for every neighbor MAP *M*_*x*_ (*x* = 2, 3, 4…). Due to the protection of the one-way hash function, even though *M*_*x*_ has its own *K’*_*MAC1*_, it still cannot produce another MAP’s *K’*_*MAC1*_. In addition, to preserve clients’ privacy and protect their identity information, we set *I*_*C1*_ = *H*(*I*_*C0*_||*K’*_*MAC1*_). That means every MAP *M*_*x*_ will obtain a number of anonymous ID numbers, one for a different client, and only this specific client *C* knows which anonymous ID number will be used. As is stated in the preliminaries section, it is assumed that each MAP has established symmetric keys with all its neighboring MAPs. After *M*_*1*_ successfully authenticates the client *C*, *M*_*1*_ encrypts the corresponding *I*_*C1*_, *K’*_*MAC1*_, and *θ*_*C*_ by using a different symmetric key shared with MAP *M*_*x*_, and sends those encrypted messages to its neighboring MAPs. Note that *M*_*1*_ sends the transfer ticket *θ*_*C*_’s original *τ*_*exp_θ*_ to *M*_*x*_ so that it will not be manipulated to illegally postpone the expiration time.

Upon receiving the message, *M*_*x*_ decrypts it using the same shared key and extracts *K’*_*MAC1*_ to prepare for future authentications with *C*. The above computations are performed by MAPs, and so no extra burdens are laid on the client. When *C* leaves *M*_*1*_ and visits *M*_*x*_, he/she executes the following handover authentication protocol:

1. Depending on the moving direction, *C* calculates a temporary MAC key *K’*_*MAC1*_ = *H*(*K*_*MAC0*_||*I*_*Mx*_) and *I*_*C1*_ = *H*(*I*_*C0*_||*K’*_*MAC1*_) in accordance with the ID number of the foreign MAP *M*_*x*_ to visit. Then, C chooses a nonce *N*_*C2*_ **∈** Z^*^_n_ and computes *N*_*C2*_*P*. Note that *C* can prepare *N*_*C2*_*P* in advance so as to reduce the workload during HAP. If *C* knows which MAP is the next one yet to visit, he/she can also pre-compute *I*_*C1*_ and *H*(*θ*_*C*_||*I*_*C1*_). Then, *C* submits *I*_*C1*_, *H*(*θ*_*C*_||*I*_*C1*_), *N*_*C2*_*P*, and the MAC value *V*_*K’MAC1*_(*I*_*C1*_, *H*(*θ*_*C*_ ||*I*_*C1*_), *N*_*C2*_*P*) to the foreign MAP *M*_*x*_. Upon receiving the message, *M*_*x*_ checks the *τ*_*exp_θ*_ of *θ*_*C*_ received from *M*_*1*_ and determines whether or not the *τ*_*exp_θ*_ has expired. Then, *M*_*x*_ verifies *H*(*θ*_*C*_||*I*_*C1*_) and the MAC value by using *K’*_*MAC1*_.
2. If the above verifications are successful, *M*_*x*_ chooses a nonce *N*_*Mx*_ **∈** Z^*^_n_, computes *N*_*Mx*_*N*_*C2*_*P* and *N*_*Mx*_*P*, and produces a formal MAC key *K*_*MAC1*_ = *H*(*K’*_*MAC1*_||*N*_*Mx*_*N*_*C2*_*P*). After that, *M*_*x*_ sends *N*_*Mx*_*P* and a MAC value *V*_*KMAC1*_(*N*_*Mx*_*P*, *N*_*C2*_*P*, *θ*_*C*_) to *C*. Upon receiving those messages, *C* computes *N*_*C2*_*N*_*Mx*_*P* and also produces a formal MAC key *K*_*MAC1*_ = *H*(*K’*_*MAC1*_||*N*_*C2*_*N*_*Mx*_*P*) and verifies the MAC value transferred from *M*_*x*_. If the verification result is positive, *C* sends *V*_*KMAC1*_(*θ*_*C*_|| *N*_*Mx*_*P*) to *M*_*x*_ to inform *M*_*x*_ he/she has successfully constructed *K*_*MAC1*_.
3. Upon receiving *V*_*KMAC1*_(*θ*_*C*_||*N*_*Mx*_*P*), *M*_*x*_ verifies the value. Since *C* is the only client that keeps *K*_*MAC1*_, it proves the validity of *C*. Now the handover authentication process is completed, and *K*_*MAC1*_ is the session key used for further communications between C and *M*_*x*_. Meanwhile, *M*_*x*_ can prepare *K’*_*MAC2*_ and *I*_*C2*_ for *C*’s next execution of *HAP*.

## 6 Security Analysis

In this section, we list the common security requirements and threats [2, 9, 11–17, 22, 28, 30–46, 44–46], along with the proof that the proposed protocol can satisfy those requirements and withstand those threats. We shall compare with some similar protocols including Li et al.’s scheme [22] and Yang et al.’s scheme [45]. Yang et al.’s scheme is a follow-up study of Li et al.’s scheme. Yang et al. also found the ticket forgery problem in Li et al.’s scheme and proposed their solution in 2015. The security comparisons are shown as Table 2.

**Table 2:** Security comparison among similar protocols.

Security requirement	Li et al. [22]	Yang et al. [45]	Ours
Mutual authentication	Yes	Yes	Yes
Privacy preservation:	No	Yes	Yes
Forward and backward security	No	Yes	Yes
Replay attack resistance	Yes	Yes	Yes
Forgery attack resistance	No	Yes	Yes

1) Mutual authentication:

Mutual authentication means that the participators of communication verify the legality of each other. In the proposed LAP, *M*_*1*_ and *C* exchange and verify each other’s ticket. The digital signature of TA can provide the legality of both *M*_*1*_ and *C*. In addition, *C* encrypts his/her ticket and the authentication information by using *M*_*1*_’s public key. Under the protection of public key cryptography, only *M*_*1*_ can decrypt this message and extract the plaintext. Therefore, it is difficult for an attacker to obtain the authentication information (e.g., *N*_*C1*_) and generate the correct reply message (e.g., *H*(*N*_*C1*_)). That means *C* can verify *M*_*1*_’s validity if *M*_*1*_ returns correct *H*(*N*_*C1*_). On the other hand, *M*_*1*_ can also verify *C* if *C* returns correct *H*(*N*_*M1*_), for only *C* can extract *N*_*M1*_ from *E*_*PC*_(*N*_*M1*_).

Before *C* moves from *M*_*1*_ to *M*_*x*_, *M*_*1*_ encrypts *K’*_*MAC1*_ by using the symmetric key *M*_*1*_*M*_*x*_. Based on symmetric cryptography, *M*_*1*_ and *M*_*x*_ can achieve mutual authentication with each other. Since the client only C has the necessary information for the construction of *K’*_*MAC1*_, *M*_*x*_ can authenticate *C* by checking the MAC value based on *K’*_*MAC1*_ during HAP. In the meantime, *C* can authenticate *M*_*x*_ if *M*_*x*_ replies with the MAC value based on correct *K*_*MAC1*_, for only *M*_*x*_ is capable of constructing the correct *K*_*MAC1*_.

2) Privacy preservation:

In the proposed protocol, *I*_*C*_ is only used when *C* launches the login request, whereas *I*_*Ck*_, which is for the *k*-th handover, is composed of a number of nonces provided by *C* and the MAPs that *C* roams through. In addition, *I*_*C*_ is encrypted by *P*_*M1*_ when the message is transmitted. That means two things: firstly, only *M*_*1*_ can obtain *I*_*C*_, and an interceptor cannot extract *I*_*C*_ from the encrypted message; secondly, *M*_*1*_ cannot track *I*_*Ck*_ because it is always re-constructed by a couple of nonces provided respectively by *C* and the current MAP when *C* is roaming to a new MAP. Besides, instead of the plaintext *θ*_*C*_, we use *H*(*θ*_*C*_ ||*I*_*Ck*_) in HAP. That means it is hard to extract *θ*_*C*_. Even when an adversary fortuitously obtains *C*’s *θ*_*C*_ in plaintext, they still have to intercept all possible *I*_*Ck*_ and compute *H*(*θ*_*C*_ ||*I*_*Ck*_) if he/she wish to track down *C*.

3) Forward and backward security:

To satisfy this requirement, we have to prove that, should an adversary somehow acquire a secret key for a certain communication session, the adversary has no way to derive the secret keys for the previous and following sessions. In the proposed scheme, the *K*_*MACk*_ for the *k*-th handover includes a couple of nonces respectively provided by *C* and the current MAP. Those nonces are protected by the elliptic curve discrete logarithm problem (ECDLP) and elliptic curve diffie-Helman (ECDH). That means given {*N*_*Mx*_*P*, *N*_*Ck*_*P*} **∈** *E*(*G*), {*N*_*Mx*_, *N*_*Ck*_}**∈** Z^*^_n_, and *P* **∈** *G*_*q*_, it is intractable to derive *N*_*Mx*_*N*_*Ck*_*P*. Therefore, even if *N*_*Mx*_*P* and *N*_*Ck*_*P* are intercepted by an adversary, the adversary still cannot derive the MAC key *N*_*Mx*_*N*_*Ck*_*P*. Furthermore, those nonces are randomly generated, so an adversary cannot derive a new MAC key should the adversary be able to break an old MAC key. In addition, since the *k*-th secret key is constructed from the collision-free one-way hash function of the (*k-1*)-th secret key, an adversary cannot derive any previous secret keys from it. To conclude, the proposed protocol guarantees perfect forward /backward security.

4) Replay attack resistance:

An adversary may eavesdrop some messages during authentication sessions and replay these messages in the future in an attempt to get authenticated and successfully access the network as a client. Similarly, an attacker may attempt to gain the client’s trust as a MAP. To protect clients and MAPs from reply attacks, we set two nonces in LAP and HAP. In LAP, the nonces *N*_*C1*_ and *N*_*M1*_ are randomly generated by *C* and *M*_*1*_ respectively and protected by public key cryptography. An adversary cannot succeed in being authenticated by replaying the message because *C* and *M*_*1*_ discard those nonces once the LAP is completed. Besides, since we use public key cryptography and one-way hash function to protect the nonces *N*_*C1*_ and *N*_*M1*_, the adversary cannot decrypt the messages and thus cannot launch a successful replay attack when the LAP is ongoing.

In HAP, the private MAC key and nonces protect the client and MAP from replay attacks. What the attacker has in hand are only the old nonces and an old MAC value recorded, which cannot pass a new authentication procedure. Even if the attacker should come by some new nonces, there is still no way they can compute the correct MAC value in the absence of the private MAC key. In conclusion, both our LAP and HAP can resist the replay attack.

5) Forgery attack resistance:

In LAP, TA’s digital signature ensures that both the client’s ticket and the MAP’s ticket are valid and unmodified. In HAP, since the MAP that *C* is leaving *M*_*1*_ for has obtained the correct information of *θ*_*C*_ from *M*_*1*_, *C* cannot forge a fabricated *θ*_*C*_. On the other hand, since the MAC value is different for each round-trip, we can ensure the integrity of these messages and thus guarantee that our HAP can resist attacks from outside. Any unofficial modification to the content of message will result in an incorrect MAC value due to the lack of the MAC key and will be identified.

## 7 Performance Analysis

In this section, we shall analyze the performance of the proposed handover authentication scheme by showing how it compares with some similar protocols including EAP-TLS [47], Li et al.’s scheme [22], and Yang et al.’s scheme [45]. EAP-TLS is a popular authentication protocol for IEEE 802.11-based wireless networks and represents the multi-hop handover authentication approach. Note that to keep real-time applications going and to provide better user experience, the overall handover latency should not exceed 50*ms* [2].

### 7.1 Computation Cost

The computation cost represents the processing delays of the cryptographic operations on both the client side and the MAP side. These operations include encryption using public key (*T*_*E*_), decryption using private key (*T*_*D*_), generation of digital signature (*T*_*sig*_), verification of digital signature (*T*_*ver*_), computation of MAC value (*T*_*MAC*_), computation of hash value(*T*_*H*_), and computation of point multiplication (*T*_*pmul*_). To be fair, we used the same public key cryptographic system—RSA-1024 as the RFC 5216 suggested for *T*_*E*_, *T*_*D*_, *T*_*sig*_, and *T*_*ver*_.—to run all the schemes compared. We learned the authentication latencies of *T*_*E*_, *T*_*D*_, *T*_*sig*_, *T*_*ver*_, *T*_*MAC*_, and *T*_*H*_ from Long and Wu’s experimental results [48]. However, Long and Wu’s experimental results do not include the latency of *T*_*pmul*_. Although they did provide ECDSA’s signature latencies, those operations are way too complex, so the results cannot translate to the latency of *T*_*pmul*_. Therefore, we turned and referred to other studies concerned to obtain the ratio of ECC to RSA. Since the decryption of ECC only involves a point multiplication and a point subtraction, we can say that the cost of time for the decryption of ECC is almost the same as that of *T*_*pmul*_. According to Ariffin and Mahad’s study, the time cost of decryption at a ratio of ECC-128 to RSA-1024 is approximately 0.0113 (0.770s: 68.042s per 625 blocks) [49]. We can then translate this to an approximated *T*_*pmul*_ time cost of 0.376*ms* (33.3*ms*×0.0113). Please note that in Long and Wu’s experiment RSA used a short-length public key and a long-length privacy key to expedite the process of public key operations (e.g., *T*_*E*_ and *T*_*ver*_), and the ratio of *T*_*D*_ to *T*_*E*_ was approximately 23.45. Because we use the public key operations as the intermediary value, the time cost of *T*_*pmul*_ may be overestimated. To clearly see how the schemes compared in terms of performance, Table 3 lists the operations discussed above, the algorithms implementing the operations, time cost of these algorithms, and the numbers of different kinds of operations performed by different protocols.

**Table 3:** Performance comparison among similar protocols.

Op. (Algorithm)	Time(*ms*)	EAP-TLS	Li et al.		Yang et al.		Ours	
			LAP	HAP	LAP	HAP	LAP	HAP
*T*_*E*_ (RSA-1024)	1.420	1	2	0	2	0	2	0
*T*_*D*_ (RSA-1024)	33.30	1	2	0	2	0	2	0
*T*_*sig*_ (RSA-1024)	33.30	1	0	0	1	0	0	0
*T*_*ver*_ (RSA-1024)	1.420	3	2	0	2	1	2	0
*T*_*MAC*_ (HMAC)	0.015	0	1	6	1	4	0	5
*T*_*H*_ (SHA-1)	0.009	3	0	0	0	0	6	3
*T*_*pmul*_ (ECC-128)	≈ 0.376	0	0	0	0	0	0	2
Total computation cost (*ms*)		72.307	72.295	0.090	105.595	1.480	72.334	0.854
Number of transmissions		9	6	3	5	3	6	3
Authentication latency (*ms*)		72.307+9*dh*	72.295+6*d*	0.09+3*d*	105.595+5*d*	1.48+3*d*	72.334+6*d*	0.854+3*d*

Because the first *T*_*pmul*_ can be performed in advance (e.g., *N*_*M*_*P* and *N*_*C2*_*P*), the number of *T*_*pmul*_ is cut down from two to one for the client. Similarity, the MAP also only needs to perform one *T*_*pmul*_. That means the total number of *T*_*pmul*_ is only two in HAP. According to Table 2, the total computation cost of the proposed scheme in LAP (72.334*ms*) is slightly higher than those of EAP-TLS (72.307*ms*) and of Li et al. (72.259*ms*) but significantly lower than Yang et al.’s scheme (1*05*.*595ms*). However, since the client only needs to do LAP one time and it lasts until the transfer ticket expires, the impact of a long LAP processing time is relatively small. More importantly, the computation cost of the proposed scheme in HAP is only 0.854*ms* and significant lower than Yang et al.’s scheme. Although the proposed HAP is slower than Li et al.’s scheme, the price is paid to mend the security flaws in Li et al.’s protocols mentioned earlier, and we think it is worth the while. With the overall handover latency kept under 1*ms*, the proposed scheme still shows pretty good efficiency.

### 7.2 Communication Cost

The communication cost is estimated in accordance with the number of message transmissions between a MAP and a client during LAP or MAP because the transmissions are what cause the communication delays. The notation *d* stands for the average delay of a transmission made across one hop, and *h* is the number of hops between the client and the verifier. Because EAP-TLS is the only scheme in the comparison that requires a client to communicate with the EAP server, which is always multi-hops away, during the handover process, the parameter *h* is applicable to only EAP-TLS. The results show that the number of transmissions made between the client and the MAP in the proposed HAP is equal to those of Li et al.’s and Yang et al.’s and is smaller than EAP-TLS. In other words, judging by communication cost, the proposed scheme performs just as well as Li et al.’s scheme.

## 8 Conclusion

In this paper, we have proposed fast handover authentication protocols based on ticket for wireless mesh network. Not only does the proposed protocol satisfy all the essential handover security requirements, but it also preserves the privacy of the client. The results of our security analysis and performance comparison show that the proposed protocol is superior to the other protocols of the kind. Even though the proposed HAP is slightly slower than Li et al.’s work, the security level is significantly higher. Moreover, like the proposed protocol, Yang et al.’s protocol is also the product of a follow-up study meant to fix the security problems of Li et al.’s work, but our new protocol turns out to be both faster and more secure. Besides, in our new design, no complicated computations need to be done on the client’s side, and therefore our new protocol is especially suitable for applications in wireless mesh networks where the mobile devices have limited computation power. Our future research will continue to focus on the development of authentication protocols that are more efficient, more reliable, and more user-friendly.
